# Local structure of human hair spatially resolved by sub-micron X-ray beam

**DOI:** 10.1038/srep17347

**Published:** 2015-11-30

**Authors:** Vesna Stanić, Jefferson Bettini, Fabiano Emmanuel Montoro, Aaron Stein, Kenneth Evans-Lutterodt

**Affiliations:** 1 Brazilian Synchrotron Light Source, CNPEM, SP 13083-970, Brazil; 2 Brazilian National Nanotechnology Laboratory, CNPEM, SP 13083-970, Brazil; 3 Center for Functional Nanomaterials, Brookhaven National Laboratory, Upton, NY 11973, USA; 4 National Synchrotron Light Source, Brookhaven National Laboratory, Upton, NY 11973, USA

## Abstract

Human hair has three main regions, the medulla, the cortex, and the cuticle. An existing model for the cortex suggests that the *α*-keratin- based intermediate filaments (IFs) align with the hair’s axis, but are orientationally disordered in-plane. We found that there is a new region in the cortex near the cuticle’s boundary in which the IFs are aligned with the hair’s axis, but additionally, they are orientationally ordered in-plane due to the presence of the cuticle/hair boundary. Further into the cortex, the IF arrangement becomes disordered, eventually losing all in-plane orientation. We also find that in the cuticle, a key diffraction feature is absent, indicating the presence of the *β*-keratin rather than that of the *α*-keratin phase. This is direct structural evidence that the cuticle contains *β*-keratin sheets. This work highlights the importance of using a sub-micron x-ray beam to unravel the structures of poorly ordered, multi-phase systems.

While Human hair has been studied by x-ray diffraction and electron microscopy for many years^1^,^2^,^3^,^4^,^5^,^6^,^7^,^8^,^9^, a complete picture has been elusive because hair is weakly scattering, disordered, and heterogeneous. Human hair is a hierarchical structure, largely constituted of intermediate filaments (IFs) that in turn are made up of keratin molecules^1^,^10^. Hair has three main regions: (1) the medulla on the central axis, (2) the cortex, and, (3) the cuticle at the exterior of the hair, (Fig. 1A,B). The medulla mainly is filled with a keratin matrix partially composed of IFs that are spatially and orientationally disordered. The majority of the cortex has a dense packing of IFs aligned along the axis of the hair, but are fluid-like in two dimensions in the plane perpendicular to the hair’s axis. The cuticle is well organized in a layered 1D-ordered structure.

Fraser and colleagues proposed a molecular structural model of *α*-keratin and the IF in 1959 and 1964^10^,^11^, but there is still controversy about the exact structure of the IFs that are a major component of hair^2^,^3^,^12^. In most x-ray measurements, many hair fibers were bundled together so as to assure significant signal-to-noise ratio^2^,^7^,^13^. The advent of high brightness synchrotron sources has enabled the usage, in a few experiments, of an x-ray micro-beam on single strands of hair. However, given the typical sizes of x-ray beams (over 2 microns) relative to the diameter of hair (60–140 microns), and the scattering geometry (x- ray perpendicular to hair’s axis), it was difficult to isolate scattering from the different regions^5^,^12^,^14^,^15^,^16^.

To spatially resolve the hair’s macro regions, we employed a sub-micron x-ray- diffraction beam of 300 nm in the high-resolution scanning direction, and 20 *μ* in the orthogonal direction. The samples were cross-sectional slices of hair of 30 *μ* thickness, with a diameter 80 *μ*; they were studied with the x-ray beam || and ⊥ to the hair’s axis.

Our main discussion here will focus on the results of high-resolution SAXS/WAXS. However, we also present our STEM imaging that is complementary to, and provides a context for, the discussion of the SAXS/WAXS data. Further details on the STEM imaging is given at the end of the discussion section.

We note that hair and wool have been extensively studied by electron microscopy and one of the first high-resolution electron microscopy studies was reported by Rogers 1959^17^,^18^, clearly showing the packing arrangement of the IFs in the cortex and cell membrane complex in the cuticle. More recent results have been obtained by TEM and tomography studies^8^,^19^.

## Results and Discussion

### Small angle x-ray scattering

**Figure 1:**
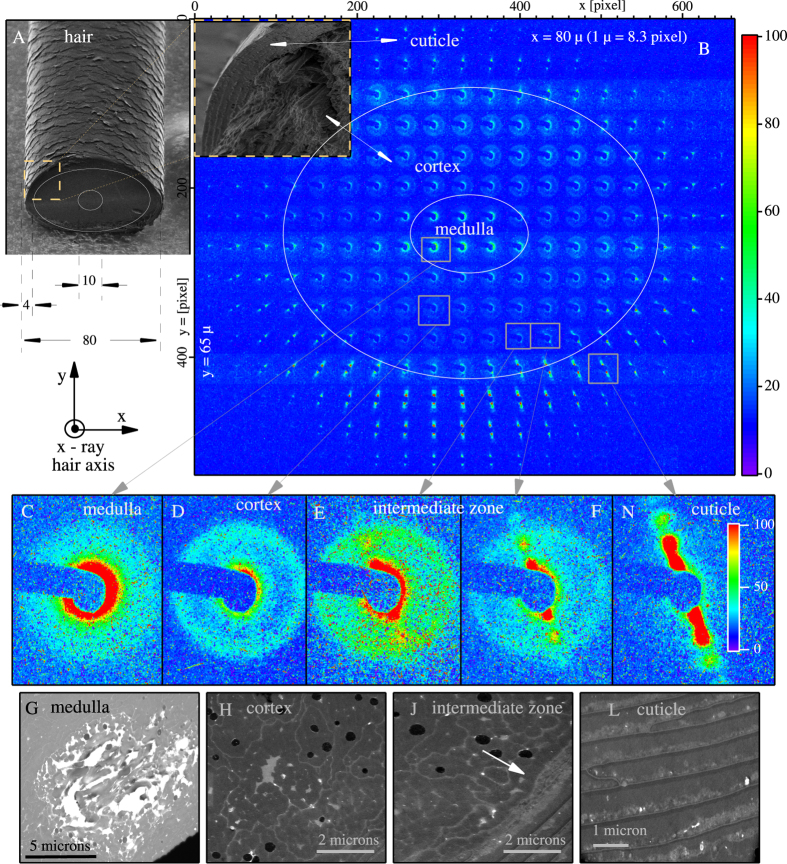
SEM image of a human hair fiber is shown in (**A**) with schematic overlays on to the cut face of the hair fiber to emphasize the relative size of the hair and the main hair regions. In (**B**), we spatially position the SAXS diffraction patterns relative to each other, so that the locations match the position of the x-ray beam on the sample, thus generating a SAXS “map” of human hair. This map allows us to see the spatial variation of the SAXS patterns from a 30-micron- thick cross-section of hair. The incident x-ray beam is 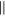 to the hair axis. SEM zoom of selected cross section from (**A**) is overlapped to the SAXS map showing the real-space cross section of the cuticle and cortex. Magnified views of the characteristic SAXS patterns are shown in (**C**) for the medulla, (**D**) for the cortex, and in (**N**) for the cuticle. (**E**,**F**) are for the newly discovered intermediate zone. Corresponding Scanning Transmission Electron Microscopy (STEM) images shown in (**G**,**H**,**J**,**L**).

In Fig. 1B, we show a representation of the diffraction data obtained from the cross-section of a hair fiber, wherein we spatially positioned the Small Angle X-ray Scattering (SAXS) part of each diffraction pattern as it was taken, so allowing us to see the evolution of the IF structural arrangements within each region of hair. We superimposed the boundaries of the different regions that are identified by the SAXS pattern. The magnified images of the characteristic SAXS patterns for the medulla, cortex and cuticle are shown respectively in Fig. 1C,D and 1N. In Fig. 1E,F, we show a SAXS diffraction pattern for a previously unidentified region, an “aligned cortex” region, which we elaborate on below. Complementary Scanning Transmission Electron Microscopy (STEM) images for these regions are shown in 1G, 1H, 1J and 1L.

Both the SAXS data and the STEM images were obtained from different slices from the same hair strands, but prepared differently for the different methods, (see method section). We emphasize that we present the STEM images to give an indication of the real-space morphology of a given hair region from which the diffraction data is obtained, to help the reader interpret the observed diffraction pattern. The STEM images are discussed later.

The characteristic SAXS for the medulla region, shown in Fig. 1C, is a symmetric pattern that is monotonically decreasing in intensity with momentum transfer. The matching real-space image, 1G, shows that the medulla is spatially and orientationally disordered, viz., consistent with the SAXS pattern. The characteristic SAXS for the cortex in Fig. 1D, shows that intensity does not decrease monotonically, but instead has an isotropic ring at 94 Å, representing the averaging of randomly oriented nearest-neighbor correlations between IFs. The matching real- space electron-microscopy image, 1H, shows the cortical cells (dark grey contrast) and the random network of walls between cortical cells (light grey contrast). Electron microscopy does not have the spatial resolution to show individual IFs. The cuticle (Fig. 1N,L) is made up of macro layers of about 0.5 *μ* thick as is shown in the real-space electron microscopy image in Fig. 1L, with a total thickness of cuticle, in our case, of about 3–4 *μ*. Here, the SAXS pattern in Fig. 1N shows an array of x-ray diffraction peaks typical of a layered structure aligned with the walls of the hair. While the layering observed in both the electron microscopy and the SAXS data are orientationally aligned, in this experiment the two techniques have mutually exclusive spatial-resolutions.

We now address the newly observed “aligned cortex” region. As we move the x-ray beam from deep inside the cortex towards the cuticle, the SAXS pattern changes from an isotropic 94 Å ring with uniform intensity around the ring (Fig. 1D), into one in which the intensity becomes anisotropic, as shown in Fig. 1E. The evolution of the diffraction pattern from Fig. 1D to Fig. 1E is a continuous function of position. Our interpretation of the intensity of the anisotropic ring is that the angular- probability distribution of next nearest neighbor bonds is influenced by their proximity to the cuticle-cortex interface. As we continued to move the x-ray beam closer to the interface, a new set of peaks appeared, consistent with the layering (210 Å and 111 Å), shown in Fig. 1F. These new peaks are precursors to the structure in the cuticle, but coexist with the 94 Å ring with an anisotropic intensity distribution.

**Figure 2:**
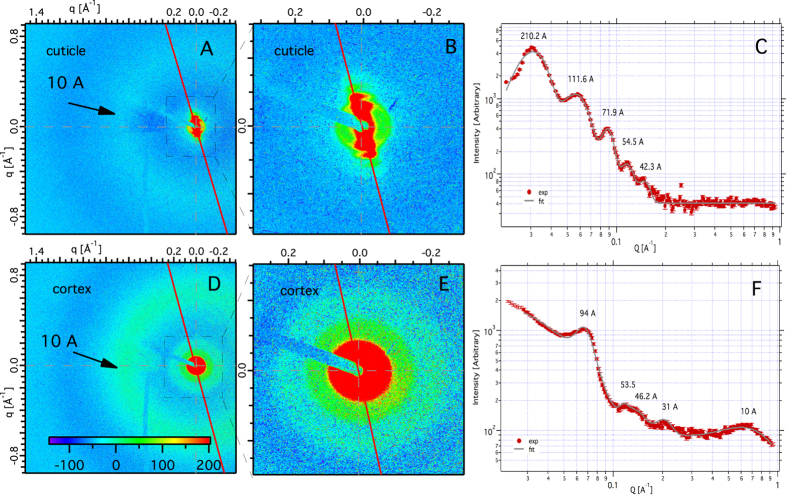
A detailed comparison of SAXS and WAXS diffraction patterns of the cuticle and cortex taken with the incident x-ray beam 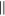 to the hair axis. The WAXS of the cuticle is shown in (**A**), and the SAXS of the cuticle is shown in (**B**). The SAXS simply is an expanded view of the interior of (**A**). Similarly, (**D**) is the WAXS of the cortex, and (**E**) is the SAXS of the cortex. In (**C**), we project the intensity along the red line in (**A**). In (**F**), we project the intensity along the red line in (**D**). The well-ordered SAXS pattern of the cuticle shown in (**B**) is anisotropic, and is characteristic of a layered structure. The SAXS features of the cortex (**E**) are isotropic, with an intense first-order 94 Å, and weaker higher orders. The broad 10 Å ring is isotropic and is barely statistically significant in the cuticle (**A**), while the same 10 Å, ring in the cortex (**D**) is more intense and is also isotropic.

In Fig. 2, we show diffraction images with better counting statistics for the cuticle (Fig. 2A,B) and cortex (Fig. 2D,E). We observed that, in addition to the primary ring at 94 Å, there are weaker, broader rings at 53.5 Å, 46.2 Å and 31 Å. This is consistent with 2D fluid-like disorder of close packed aligned IFs, probably with a short-range hexagonal, para-crystal arrangement^2^,^20^,^21^.

Line projections of the cuticle and cortex region are shown in Fig. 2C,F. In the cuticle, we observe up to 5 diffraction peaks with d spacings of 210.2 Å, 111.6 Å, 71.9 Å, 54.5 Å, 42.3 Å, which indicates a rearrangement of the keratin molecules not typical of the *α*- phase. A well-ordered, layered structure is more indicative of *β* keratin, as we confirm below.

**Figure 3:**
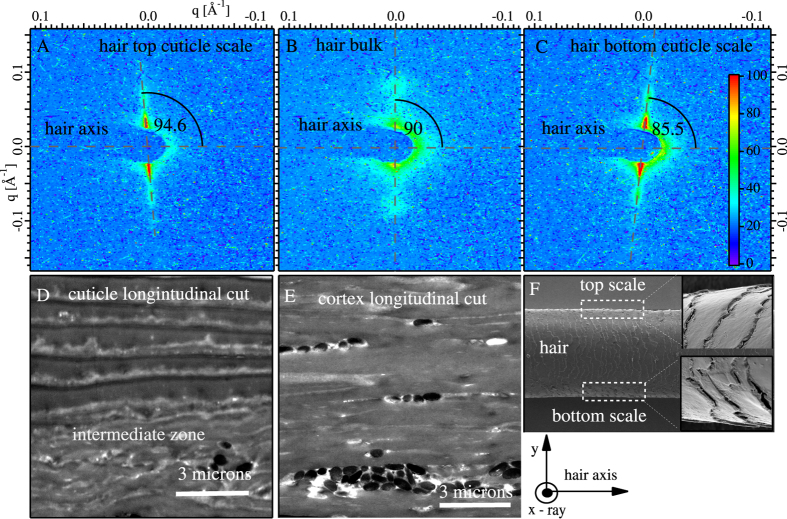
The SAXS patterns taken on a single hair with the incident X-ray beam ⊥ to the hair axis is shown in (**A**) to (**C**). (**A**) is for the top of the hair in the cuticle, (**B**) is largely the cortex region and (**C**) for the bottom edge of the hair in the cuticle region. Corresponding real- space STEM images of hair cut longitudinally are shown in (**D**) (cuticle) and (**E**) (cortex) with no staining or coating. In (**D**), we again observe the intermediate zone. (**F**) shows SEM images of the hair and the detail of the cuticle shows that the’s scales are oriented with an angular offset with respect to the hair’s axis. The angular offset between the hair’s axis and the cuticle scales in (**F**) is the same angular offset we observe in reciprocal space between (**A**,**B**), or (**B**,**C**).

To compare with previous publications, we took diffraction images with the beam ⊥ to the hair axis, as depicted in Fig. 3A–C. STEM images of the cortex and cuticle, cut in a longitudinal plane are shown respectively, in Fig. 3D,E. The SAXS from the cortex (Fig. 3B) shows two peaks at ±*q*_0_ on an axis that is ⊥ to the direction of the hair’s axis. Relative to this axis, the cuticle peaks that are at the top of the hair (Fig. 3A) and bottom of the hair (Fig. 3B) are offset, respectively, by 4.6 ± 0.1 degrees and −4.5 ± 0.1 degrees. These angular offsets have been correlated with the orientation of the cuticle scales relative to the hair axis, as viewed from the ⊥ direction (SEM image in Fig. 3F). Other than these angular offsets, the layer spacing of the cuticle in Fig. 3A,C are consistent to those observed in Fig. 2B. Clearly, the SAXS data from the 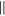 and ⊥ directions show (Fig. 1N,A,C) that the cuticle is layered on the 210 Å length-scale, while electron microscopy (Fig. 1L) shows that it is layered on the 5000 Å length scale.

### Wide angle x-ray scattering

**Figure 4:**
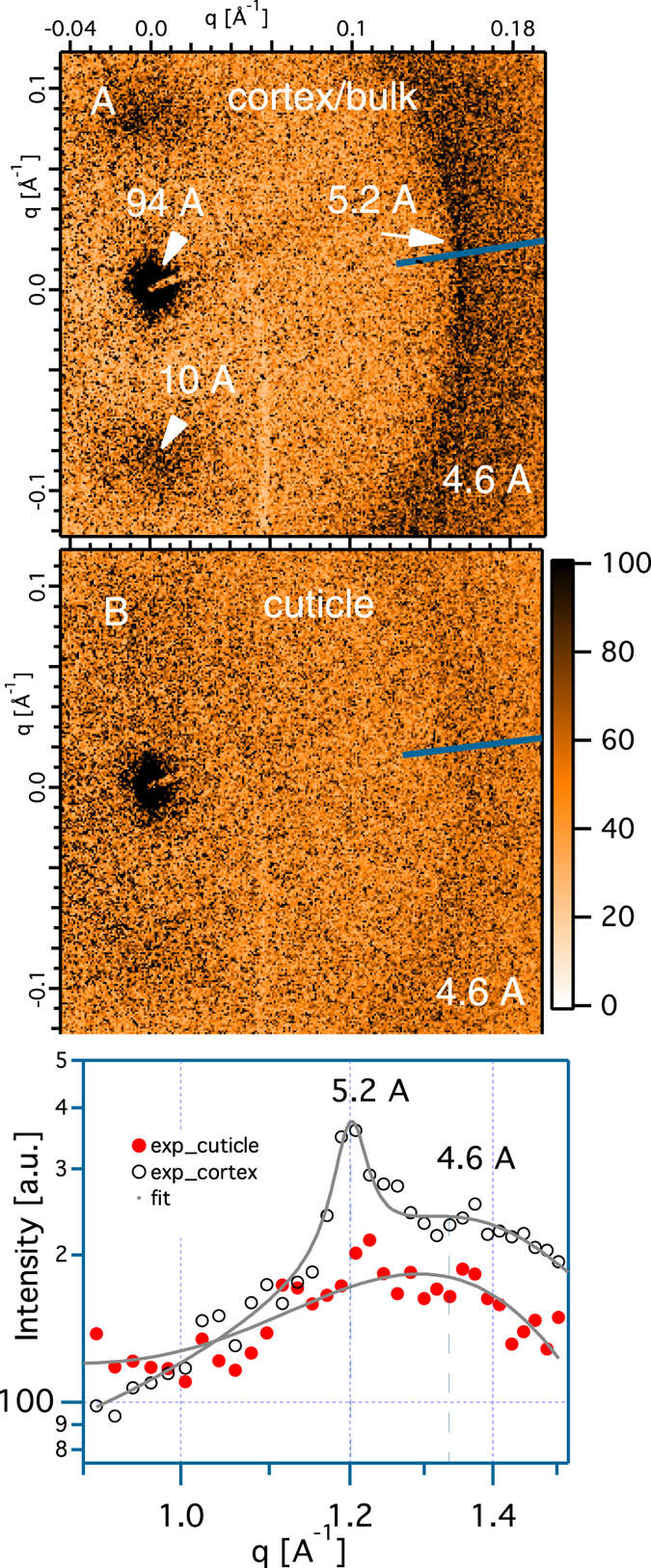
Comparison of the wide angle x-ray scattering of cortex shown in (**A**) and cuticle shown in (**B**) measured with x -ray beam ⊥ to the hair’s axis. (**C**) showing the line projections of the integrated intensities for the peak 5.2 Å and the peak 4.6 Å of the two respective regions.

As shown in Fig. 4, wide angle x-ray scattering (WAXS) with the x- ray beam ⊥ to the hair axis, reveals structural information internal to the IFs. Three features are shown; a broad anisotropic 10 Å peak, a broad isotropic 4.6 Å ring, and a sharp arc at 5.2 Å. In Fig. 2A,D where the incident x-ray beam is 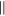 to the hair’s axis, we can compare the 10 Å ring in the cuticle, and cortex. Clearly, the 10 Å feature is stronger in the cortex. The 10 Å ring reflects the correlations between keratin-coiled coils^11^,^21^,^22^.

Comparing the WAXS patterns of the cortex (Fig. 4A) and the cuticle (Fig. 4B) more carefully as is done in Fig. 4C, we find that the sharp 5.2 Å peak, which is characteristic of the *α* keratin, is present in the cortex but absent in the cuticle. In contrast, the broad 4.6 Å feature simply is less intense in the cuticle, but its width is unchanged between the two regions. The absence of the 5.2 Å peak, and a weakening of the 10 Å peak are characteristic of a transition of *α* to *β* keratin^23^,^24^. These results corroborate suggestions from Raman scattering^25^, viz., that the cuticle has *β* keratin. Considering the layered morphology of the cuticle, and the known propensity of *β* keratin to form layered structures, it is reasonable to assume that the cuticle contains *β* keratin sheets.

An existing model on cuticle structure obtained primarily from SAXS data is reported in the literature^5^,^12^, but this model is incomplete because it does not incorporate WAXS measurements. In this model it is proposed that the layered diffraction peaks come only from the complex of the cuticle membrane and that the rest of the cuticle is amorphous^5^,^12^. The WAXS data we have presented here indicates that a significant fraction of the cuticle is constituted of *β* keratin. While a smaller x-ray beam and more study is needed to resolve the structure of cuticle sub-regions, such as the cuticle membrane complex, exocuticle or endocuticle, our results suggest that the previous model based only on SAXS data taken in orthogonal geometry needs to be reconsidered.

### Scanning Transmission Electron Microscopy

As mentioned above, the STEM measurements were primarily included to show the real space morphology of the different macro regions of hair. Below, we discuss some details of the STEM images.

#### Cross sections

In Fig. 1, we show the cross sections of the cuticle, cortex, and medulla. Figure 1G shows the medulla with a visible macro porous section. This empty porous part is a result of preparing the sample. The keratin matrix liquid, that normally fills the porous part, has evaporated during the preparation of the 60-nm thick sample slice. Figure 1H shows the cortex, the main visible components of which are 500-nm macro fibrils and circular black objects which are the hair’s pigment, melanin. There also are some gray zones, with different contrast due to the different orientation of IFs in the zones^8^,^17^. In Fig. 1J we show the intermediate zone of the cortex/cuticle, wherein we can observe the alignment and accumulation of the cell walls oriented by the hair’s curvature. Finally, in Fig. 1L, we show the cuticle’s macro layers with very high contrast in the layers. Different gray contrast and stratification is visible. It is known that hair contains and accumulates minerals in the cuticle, and we suspect that the minerals are represented by some of the lighter contrast, compact shapes in Fig. 1L, but these are beyond the scope of this work.

#### Longitudinal sections

Figure 3D to 3E are the STEM images of slices cut longitudinally along the hair’s axis. The intermediate zone at the cuticle/cortex interface clearly is visible (Fig. 3D). We also emphasize here that the longitudinal section (Fig. 3E) of the cortex looks quite different from its cross-section (Fig. 1H) The macro-fibrils clearly are visible in cross section (Fig. 1H), but not as obvious as in the longitudinal section.

In the longitudinally cut cortex region (Fig. 3E) we see that hair pigments (melanin) with sizes of about 1- to 2-microns are grouped in long clusters and align with the long hair’s axis.

### Conclusions

By using a sub-micron x-ray beam, we were able to resolve the structure of the regions of human hair. Within the cortex, we found a new region near the cuticle where the local arrangement of the IFs are oriented by the wall. Additionally, we present direct structural evidence that the cuticle of human hair contains *β*-keratin sheets. This study highlights the importance of a sub-micron x-ray beam in unraveling the structures of poorly ordered, biological multi-phase systems. We anticipate that smaller x-ray beams will improve our understanding of hair, and similarly complex systems.

## Methods

### Sample preparation

Long hair strands were cut from the back of the head from males between 25–30 years old of Japanese origin. The hair is virgin and never had been treated with any reagents except for a normal wash. All slices for the experiments are cut far from follicles or the end of the hair, in order to get the healthy part of the hair.

For embedded hair samples, long hair fibers were placed horizontally in a mold on a flat silicon-wafer that keeps the hair centered in the middle of the mold and leveled. No stretching, pressure, or deformation is applied to the hair. Resin (Pelco Eponate 12 with BDMA) is poured over the hair and allowed to solidify. The embedded samples were left at room temperature to solidify. Just before the measurements, the hair was sliced to 60 nm thickness using an ultramicrotome (model: RMC Boeckeler PowerTome X) for STEM measurements, and to 30 microns thickness by microtome (model: Leica RM 2135) for x-ray measurements. We used a MT-Diamond blade made by Pelco with a knife angle of 45 degrees All samples are examined by optical- and SEM-microscope before taking the x-ray measurements.

### X-ray measurements

X-ray measurements were performed in air, at room temperature, with the relative humidity around 48%, at the X13B beamline at the National Synchrotron Light Source at Brookhaven National Laboratory. This beamline is an undulator-based beamline with a gravity-fed water-cooled monochromator for enhancing the beam’s stability. The monochromatized photons are incident on a Kinoform lens, which creates a focused x-ray beam of 300 nm by 20 *μ* on the sample. The sample is positioned with a six degree angle of freedom stage (Smarpod 110.45), which, in turn, is placed on top of a high precision rotary air- bearing stage (MICOS UPR-120Air). Consequently, the sample can be translated in three degrees of freedom (3 DOF) with 100-nm resolution, and rotationally oriented with 0.001 degree resolution in with 3 DOF. Downstream of the sample is a beam-stop constructed of a 4-mm-long cylinder of Tungsten with diameter 500 microns. Downstream of the beam stop, there is a choice of two detectors, a visual detector that is used to position the hair in the direct x-ray beam, and a large area X-ray CCD from Princeton Instruments for quantitatively measuring the X-ray diffraction data. The visual detector system consists of a 100- micron thick YAG crystal, viewed by a microscope objective. The CCD has a diagonal dimension of 165 mm, with a 56-micron pixel size.

### Relevance of the micro-beam SAXS methodology

Hair has a heterogeneous arrangement of sub-components with slightly different structures. A traditional SAXS pattern, with a large x-ray beam, results in a sum of diffraction SAXS patterns, weighted by the volume fraction of the different regions. In the case described here, where the volume of the cortex is much larger than that of the cuticle, the traditional SAXS approach in the past has obscured the cuticle’s signal. By providing an x-ray beam that was small enough to select just the cuticle region, we were able to observe the absence of the relatively weak signature of the a-keratin phase in the cuticle region.

### STEM and SEM measurements

The SEM and STEM imaging were performed with Scanning Electron Microscope FEI Quanta 50F. STEM images are taken on hair cross-sections (60 nm thickness) without any staining or coatings.

### Ethics approval

Several participants were interviewed, and signed consent was obtained from the participants for the use of the samples. In total, 3 samples of hair were obtained from the men, ranging in age from 20 to 30 years old. The samples have no personal identification and the experimental protocols were approved by Brookhaven National Labs, Photon Sciences Safety Committee and the Office of Research Administration, which processes all human subject protocols. Protocols are in according with the “Resolution 199/96 on research involving human subjects” as well as the guideline of Brazilian National Health Council according to the Decree n: 93933 for research involving human subjects.

## Additional Information

**How to cite this article**: Stanić, V. *et al.* Local structure of human hair spatially resolved by sub-micron X-ray beam. *Sci. Rep.* **5**, 17347; doi: 10.1038/srep17347 (2015).

## References

[b1] Pauling L. & Corey R. B. The structure of hair, muscle, and related proteins. PNAS 37, 261 (1951). 14834149 10.1073/pnas.37.5.261 PMC1063352

[b2] Briki F., Busson B. & Doucet J. Organization of microfibrils in keratin bers studied by x-ray scattering modelling using the paracrystal concept. Biochimica et Biophysica Acta 1429, 57 (1998). 9920384 10.1016/s0167-4838(98)00216-7

[b3] Rafik M. E., Doucet J. & Briki F. The intermediate filament architecture as determined by x-ray diffraction modeling of hard a-keratin. Biophys J. 86, 3893 (2004). 15189886 10.1529/biophysj.103.034694 PMC1304291

[b4] Merigoux C. *et al.* Evidence for various calcium sites in human hair shaft revealed by sub-micrometer x-ray fluorescence. Biochimica et Biophysica Acta 1619, 53 (2003). 12495815 10.1016/s0304-4165(02)00441-5

[b5] Kajiura Y. *et al.* Structural analysis of human hair single fibres by scanning microbeam saxs. J. Struct. Biol. 155, 438 (2006). 16774836 10.1016/j.jsb.2006.04.008

[b6] Rafik M. E., Briki F., Burghammer M. & Doucet J. *In vivo* formation steps of the hard a-keratin intermediate filament along a hair follicle: Evidence for structural polymorphism. J. Struct. Biol. 154, 79 (2006). 16458019 10.1016/j.jsb.2005.11.013

[b7] Wade M. *et al.* Investigating the origins of nanostructural variations in differential ethnic hair types using x-ray scattering techniques. International Journal of Cosmetic Science 35, 430 (2013). 23634942 10.1111/ics.12061

[b8] Harland D. P. *et al.* Three-dimensional architecture of macrofibrils in the human scalp hair cortex. J. Struct. Biol. 185, 397 (2014). 24486856 10.1016/j.jsb.2014.01.010

[b9] Parry D. A. Fifty years of fibrous protein research: A personal retrospective. Journal of Structural Biology 186, 320 (2014). 24148884 10.1016/j.jsb.2013.10.010

[b10] Fraser R. D. B., MacRae T. P. & Rogers G. E. Structure of alpha-keratin. Nature 183, 591 (1959). 10.1038/183592a0 13632803

[b11] Fraser R. D. B., MacRae T. P. & Miller A. The fourier transform of the coiled-coil model for a-keratin. Acta Crystallo- graphica 17, 813 (1964). 10.1107/s0365110x65002621 14323453

[b12] Kreplak L., Mérigoux C., Briki F., Flot D. & Doucet J. Investigation of human hair cuticle structure by microdiffraction: direct observation of cell membrane complex swelling. Biochimica et Biophysica Acta 1547, 268 (2001). 11410283 10.1016/s0167-4838(01)00195-9

[b13] Rafik M., Doucet J. & Briki F. The intermediate filament architecture as determined by x-ray diffraction modeling of hard a-keratin. Biophysical Journal 86, 3893 (2004). 15189886 10.1529/biophysj.103.034694 PMC1304291

[b14] Busson B., Engtröm P. & Doucet J. Existence of various structural zones in keratinous tissues revealed by x-ray microdiffraction. Journal of Synchrotron Radiation 6, 1021 (1999).

[b15] Bertrand L., Doucet J., Simionovici A., Tsoucaris G. & Walter P. Lead-revealed lipid organization in human hair. Biochimica et Biophysica Acta 1620, 218 (2003). 12595092 10.1016/s0304-4165(02)00538-x

[b16] Kajiura Y. *et al.* Structural analysis of single wool fibre by scanning microbeam saxs. Journal of Applied Crystallography 38, 420 (2005).

[b17] Rogers G. Electron microscope studies of hair and wool. New York Acad Sciences 83, 378 (1959). 10.1111/j.1749-6632.1960.tb40914.x 14438365

[b18] Rogers G. E. Electron microscopy of wool. J. Ultrastructure Research 2, 309 (1959). 10.1016/s0022-5320(59)80004-6 13655351

[b19] Bryson W. G. *et al.* Cortical cell types and intermediate filament arrangements correlate with fiber curvature in Japanese human hair. J. Struct. Biol. 166, 46 (2009). 19159689 10.1016/j.jsb.2008.12.006

[b20] Fraser R. B., Miller A., Suzuki E. & MacRae T. P. The quantitative analysis of fibril packing from electron micrographs. J. Mol. Biol. 9, 250 (1964).

[b21] McKinnon A. The self-assembly of keratin intermediate filaments into macrofibrils: Is this process mediated by a mesophase? Current Applied Physics 6, 375 (2006).

[b22] Watts N. *et al.* Cryo-electron microscopy of trichocyte (hard alpha-keratin) intermediate filaments reveals a low-density core. Journal of Structural Biology 137, 109 (2002). 12064938 10.1006/jsbi.2002.4469

[b23] Kreplak L., Doucet. J., Dumas P. & Briki F. New aspects of the a-helix to b-sheet transition in stretched hard a-keratin fibers. Biophysical Journal 87, 640 (2004). 15240497 10.1529/biophysj.103.036749 PMC1304386

[b24] Garson J., Doucet J., Lévêque J. & Tsoucaris G. Oriented structure in human stratum corneum revealed by x-ray diffraction. The J. of Investigative Dermatology 96, 43 (1991). 10.1111/1523-1747.ep12514716 1987295

[b25] Bito K. *et al.* Protein secondary structure imaging with ultrabroadband multiplex coherent anti-stokes raman scattering (cars) microspectroscopy. J. Phys. Chem. B 116, 1452 (2012). 22220757 10.1021/jp210914x

